# Olanzapine alters the expression of gasotransmitter-related enzymes: CBS and HO-2 in the rat hippocampus and striatum

**DOI:** 10.1007/s43440-023-00538-5

**Published:** 2023-10-24

**Authors:** Artur Pałasz, Julia Kistowska, Aleksandra Suszka-Świtek, Marek Krzystanek, Iwona Błaszczyk, Itiana Castro Menezes, Łukasz Filipczyk, Katarzyna Bogus

**Affiliations:** 1 Department of Histology, Faculty of Medical Sciences in Katowice, Medical University of Silesia, Ul. Medyków18, 40-752, Katowice, Poland; 2 Department and Clinic of Psychiatric Rehabilitation, Faculty of Medical Sciences, Medical University of Silesia in Katowice, Ul. Ziołowa 45/47, 40-635 Katowice, Poland; 3 Department of Neurosciences and Behaviour, Faculty of Medicine, University of São Paulo, Av. Bandeirantes 3900, Ribeirão Preto, São Paulo, 14049-900 Brazil

**Keywords:** Olanzapine, Hydrogen sulfide, Carbon monoxide, Gasotransmitters, Antipsychotics, Brain, CBS, HO-2

## Abstract

**Background:**

Gaseous neurotransmitters have been thought to be novel factors involved in the mechanisms of mental disorders pathogenesis for quite some time. However, little is known about the potential crosstalk between neuronal gasotransmitter signaling and neuroleptics action. The present work was, therefore, focused on gene expression of H_2_S and CO-producing enzymes in the brains of rats chronically treated with olanzapine, an atypical antipsychotic drug.

**Methods:**

Studies were carried out on adult, male Sprague–Dawley rats that were divided into 2 groups: control and experimental animals treated with olanzapine (28-day-long intraperitoneal injection, at a dose of 5 mg/kg daily). All individuals were sacrificed under anesthesia and the whole brains excised. Immunohistochemical procedure was used for histological assessment of the whole brain and for quantitative analysis of cystathionine β-synthase (CBS) and heme oxygenase 2 (HO-2) protein distribution in selected brain structures.

**Results:**

Long-term treatment with olanzapine is reflected in different changes in the number of enzymes-expressing cells in the rat brain. Olanzapine decreased the number of CBS-expressing cells and possibly reduced H_2_S synthesis in the hippocampus and striatum. The antipsychotic administration increased the number of HO-2 immunopositive cells and probably stimulated the CO production in the hippocampus.

**Conclusions:**

Modulatory effect of olanzapine on cellular mechanisms of gasotransmitter synthesis may be an alternative way of their pharmacological action.

**Supplementary Information:**

The online version contains supplementary material available at 10.1007/s43440-023-00538-5.

## Introduction

Nitric oxide (NO), carbon monoxide (CO), and hydrogen sulfide (H_2_S) form a unique group of signaling molecules called gaseous neurotransmitters or gasotransmitters [1]. Due to very low molecular weight and lipophilic properties, their small molecules can easily cross neuronal and glial cell membranes. Differently from classical synaptic neurotransmitters, they are synthesized and released only on demand, instead of previously produced and stored in the synaptic vesicular pool [1, 2]. H_2_S has a neuroprotective effect in the course of neurodegenerative diseases and may have antidepressant and anxiolytic effects, which have been tested on mouse and rat models of depression and anxiety and protect neurons against the destructive effects of oxidative stress. Studies conducted on rats given lead Pb^2+^ ions to impair memory and cause damage to the CA1 area of the hippocampus showed that the administration of H_2_S caused a significant reduction in apoptosis in the hippocampus and improved cognitive function. This demonstrates the ability of H_2_S to reverse neural tissue damage [3]. Oxidative stress may be an important factor contributing to the occurrence of psychotic disorders and the development of schizophrenia. Overexpression of HO in transgenic mice containing the glial fibrillar acidic protein (GFAP) promoter fragment showed phenotypic similarities to human schizophrenia. It has also been shown that factors associated with the development of schizophrenia, such as ischemia, the presence of pro-inflammatory cytokines, and overproduction of dopamine, induce the gene encoding heme oxygenase 1 (HMOX1), which is associated with disturbed redox homeostasis and dysfunction of mitochondria in neurons in the prefrontal cortex of patients with schizophrenia. In the prefrontal cortex of patients with schizophrenia, overexpression of HO has been revealed, which in turn may lead to impairment of mitochondrial function through inappropriate heme metabolism [4]. H_2_S enhances the induction of LTP by increasing the activity of NMDA receptors. This induction is mediated by calcium/calmodulin-dependent protein kinase II, which regulates the activity of cystathionine β-synthase (CBS), which is involved in the production of H_2_S interacting with the NMDA receptor. H_2_S induces Ca^2+^ influx into astrocytes by activating TRPA1 ion channels, which triggers the release of D-serine into the synaptic cleft. TRPA1 channels have two sensitive cysteine residues at the anion end that react with H_2_S to form a cysteine disulfide bridge, allowing the release of D-serine. This mechanism increases the activity of NMDA receptors, which leads to the facilitation of LTP induction. In addition, studies have shown that genetic disabling of CBS impairs LTP [5].

Carbon monoxide stimulates the activity of guanylate cyclase and PKG, which means that it is involved in LTP [6]. In addition, sGC stimulation increases cGMP in neurons, which has a neuroprotective effect [7]. Studies by Dreyer-Andersen et al. (2018) indicate a decrease in the level of neurotrophin 3 and an increase in neurotrophin 4 in cells subjected to CO [8]. These neurotrophins are responsible, among others, for the growth, maturation, and plasticity of synapses. However, these are only preliminary observations that must be the basis for further research into the effects of carbon monoxide on brain neuroplasticity.

Atypical antipsychotics may affect several elements of neuronal signaling machinery, including dopamine and serotonin receptors. Olanzapine, a second-generation antipsychotic agent, acts as an antagonist of brain dopaminergic receptors (D_1_–D_5_), with some affinity to others, including serotoninergic (5-HT_2A/2C_, 5-HT_3_, 5-HT_6_), α1-adrenergic, muscarinic and histaminergic [9]. Olanzapine reduces both negative and positive schizophrenia symptoms via selective silencing of mesolimbic dopaminergic neurons, without depression of striatal neuronal circuits involved in motor functions [10, 11]. Olanzapine, such as other second-generation antipsychotics, is less effective for cognitive dysfunctions, which may be associated with changes in the composition of NMDA receptors that may possibly relate to NO-dependent signaling mechanisms [12].

So far, a number of basic studies have been conducted on the effects of olanzapine on the level of expression of many intracellular factors, while some of them may significantly contribute to the pharmacological effects of long-term treatment with this antipsychotic drug. Possible direct or indirect action of antipsychotic drugs at the level of brain gasotransmitter signaling is so far completely unknown. The aim of the study was, therefore, to investigate the effect of long-term administration of olanzapine on the expression of the H_2_S, and CO-produced enzymes: cystathionine β-synthase (CBS) and heme oxygenase-2 (HO-2) in the rat brain. CBS catalyzes the first step of the transsulfuration pathway from L-homocysteine to L-cystathionine and H_2_S synthesis. The enzyme may also generate H_2_S in an alternative way by the condensation of L-homocysteine with cysteine [5]. HO-2 catalyzes the heme cleavage to form biliverdin which is converted to bilirubin and CO [6]. The obtained results may provide new information on the potential impact of new-generation antipsychotics on the gasotransmitter-dependent mechanisms of neuroplasticity. The present experimental paradigm aims to shed light on this area by determining if and how extended antipsychotic administration influences the brain synthesis of gaseous neurotransmitters.

## Materials and methods

### Animals

The studies were carried out on adult (2–3 months, 180–210 g) male Sprague–Dawley rats from the Medical University of Silesia Experimental Centre housed at 22 °C with a regular 12/12 light–darkness cycle with access to standard Murigran chow and water ad libitum. All experimental procedures were approved by the local bioethics committee at the Medical University of Silesia (agreement no 36/2012, dated 18.04.2012).

### Drug administration

Two groups of rats (*n* = 5) received control vehicle (saline) and olanzapine (5 mg/kg), respectively, by intraperitoneal injection for 4 weeks. The above-mentioned non-toxic doses of drugs were established on the basis of pharmacological standards developed in preclinical studies.

### Brain tissue collection

24 h after the last drug administration, animals were anaesthetized and perfused with 4% paraformaldehyde PBS (pH 7.2–7.4). The brains were quickly excised, postfixed, dehydrated, embedded in paraffin, and finally sectioned on the microtome (Leica Microsystems, Germany) in the coronal plane (2.00 to 1.20 mm and − 2.52 to − 3.48 mm from bregma) at 7 μm slice thickness, according to Paxinos and Watson’s The Rat Brain in Stereotaxic Coordinates [13].

### Immunohistochemistry and immunofluorescence

After tissue deparaffinization, rehydration, antigen retrieval (in low pH citric acid buffer) and blockage with 10% serum (appropriate for primary antibody), brain sections were incubated overnight at 4 °C with the following rabbit antibodies against rat CBS (1:1000, Fine Test Biotech; FNab01327), and rat HO-2 (1:500, Enzo Life Sciences; ADI-OSA-200-F). Incubation with primary antibodies was followed by the administration of biotinylated anti-goat/anti-rabbit secondary antibodies (1:200), and then an avidin–biotin–horseradish peroxidase complex (Vectastain ABC kit, Vector Labs). 3,3′-diaminobenzidine (DAB) was used to complete the reaction and visualize immunoreactive structures (cell bodies and their processes) in the whole brain. Positive cells were counted from the hippocampal and striatal areas of each section using ImageJ 1,43u software. All images were captured and proceeded with Nikon Coolpix optic systems and processed using Image ProPlus software (Media Cybernetics, Rockville, MD, USA). The same planes of the brain were chosen from each set of slides. For the calculation of CBS and HO-2-expressing cells, 5 slices (every fifth one from the series) per rat for each hippocampal and striatal slice were used. Anatomically comparable sections were analyzed and immunopositive cells were counted using Image J 1.43 u software. We counted the total number of enzymes-expressing cells in the standard frame (500 × 400 mm) for each rat (which was the sum of cells from 5 slices).

### Statistical analysis

Statistical analysis was performed using Statistica 10 (Systat software). Gaussian distribution and variance homogeneity were estimated with the Shapiro–Wilk test. The differences between groups were analyzed using the Mann–Whitney *U* test. Differences were considered statistically significant at *p* < 0.05. Data are presented as median with interquartile ranges.

## Results

### CBS

**Fig. 1:**
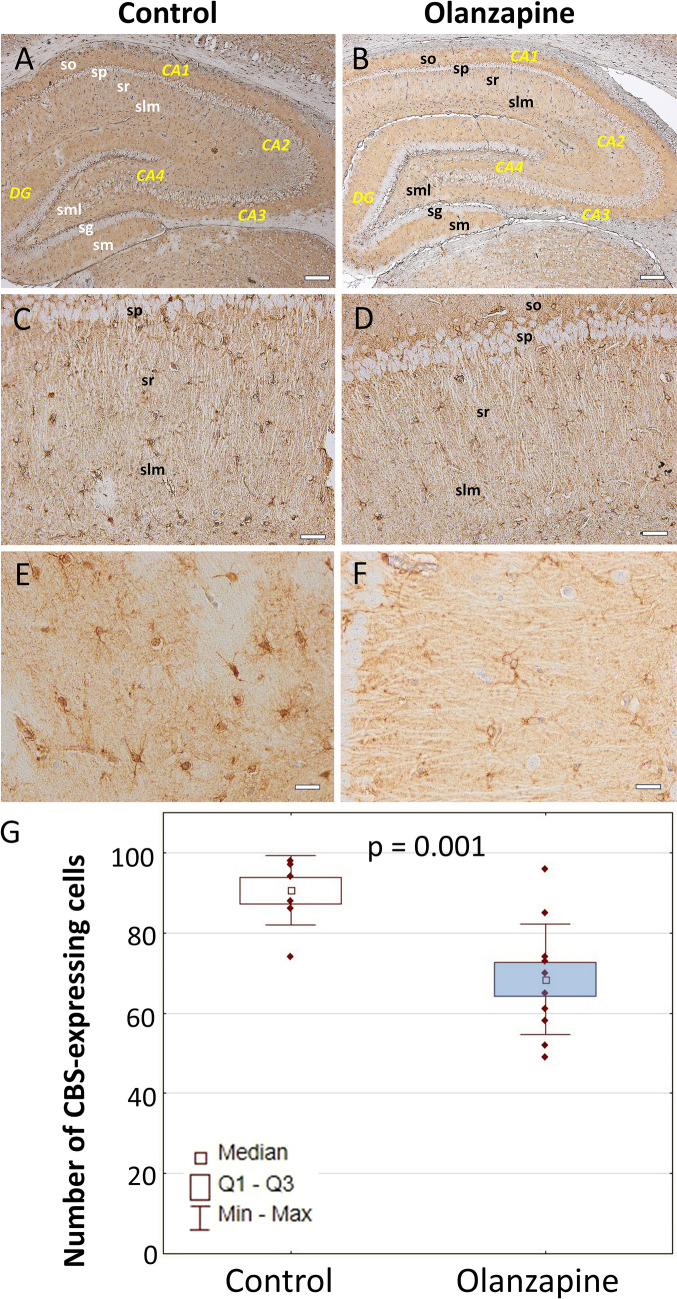
Cellular expression of CBS in the hippocampus. An overview (**A**, **B**). Area CA1 (**C**–**F**). Astrocyte-like cells in the stratum lacunosum–moleculare. DG, dentate gyrus; sg, granular layer of dentate gyrus; slm, stratum lacunosum–moleculare; sm, stratum moleculare of dentate gyrus; sml, multiform layer of the dentate gyrus; so, stratum oriens; sp, stratum piramidale; sr, stratum radiatum. The number of CBS-expressing cells in the hippocampus of control rats and animals chronically treated with olanzapine (**G**). Data are presented as median with interquartile ranges. Differences between groups (*n* = 5) were statistically analyzed using the Mann–Whitney *U* test and were considered significant at *p* <0.05. Scale bars: 200 μm (**A**, **B**), 50 μm (**C**, **D**), 20 μm (**E**, **F**)

**Fig. 2:**
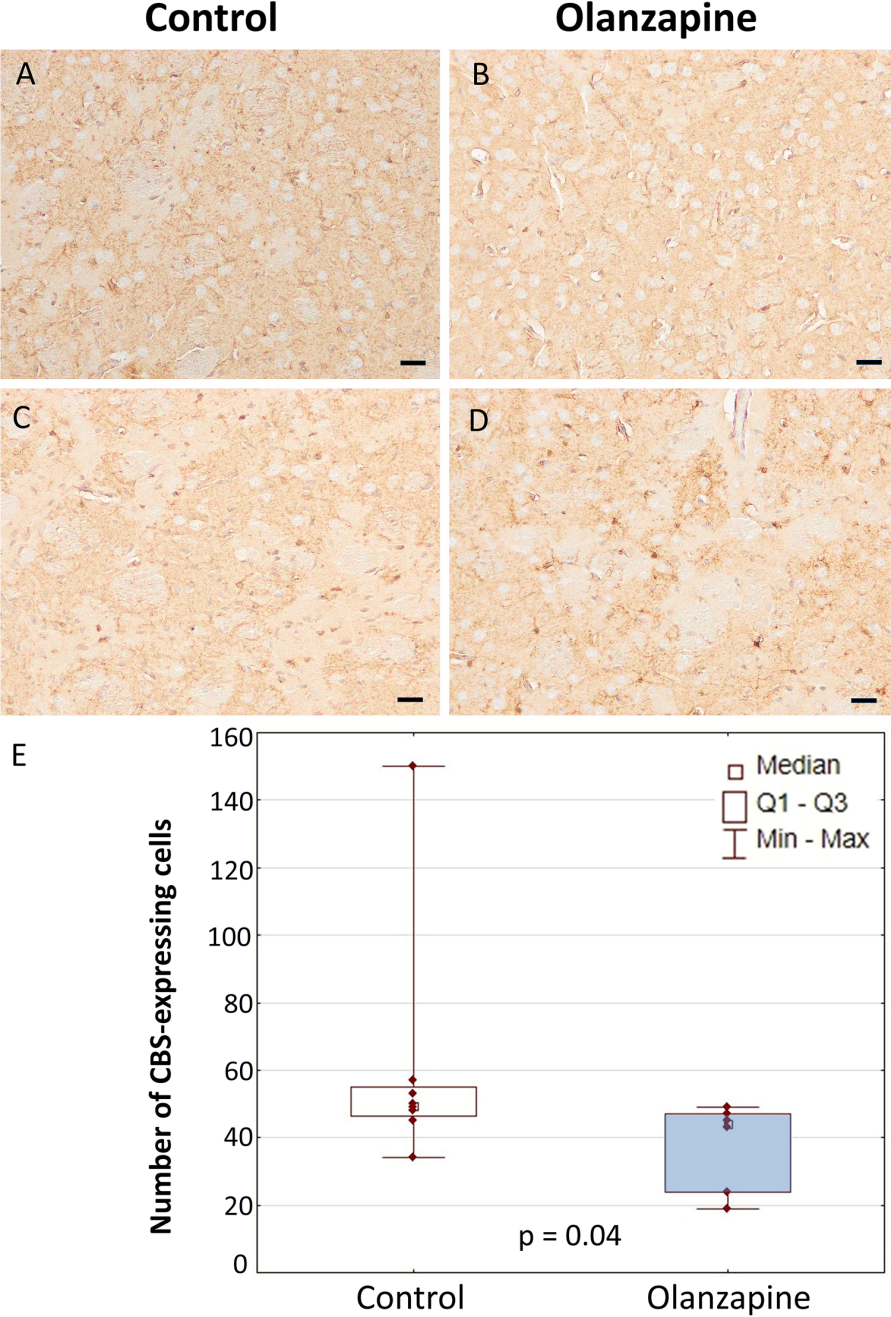
Expression of CBS in the striatum (**A**–**D**). The number of CBS-positive cells in the striatum of control rats and animals chronically treated with olanzapine (**E**). Data are presented as median with interquartile ranges. Differences between groups (*n* = 5) were statistically analyzed using the Mann–Whitney *U* test and were considered significant at *p* <0.05. Scale bars: 50 μm

Control: An overview of CBS expression conducted under lower magnification of the sample revealed the presence of numerous immunopositive astrocyte-like cells on the almost whole brain regions (Fig. 1 and Supplementary Data). White matter areas of the hippocampal formation and striatum were characterized by a particularly large number of CBS-expressing cells. Quantitative analysis of the number of CBS-positive astrocytes was performed in the stratum radiatum and stratum lacunosum–moleculare of the CA1 area of the Ammon’s horn as well as in the striatum (caudoputamen). It is worth noticing, that a few, weakly branched interneuron-like cells of the hippocampal formation scattered between CA1 pyramidal neurons also seemed to demonstrate a visible CBS expression (Fig. 1 and Supplementary Data). However, their precise identification and differentiation from astrocytes are not possible without examining the potential co-expression with, e.g., NeuN and GFAP protein or additional neuronal and glial markers. A smaller population of CBS-expressing cells with a more diffuse distribution profile was observed in the striatum (Fig. 2 and Supplementary Data). Olanzapine: The morphological profile did not differ from that observed in the control group and the expression profile of CBS-positive cells was identical (Fig. 2 and Supplementary Data). Quantitative analysis revealed a statistically significant decrease in the number of immunopositive cells compared to the control group in the whole hippocampus (*p* = 0.001, *U* = 5, *N* = 18) and the striatum (*p* = 0.04, *U* = 8, *N* = 14) (Fig. 2).

### HO-2

**Fig. 3:**
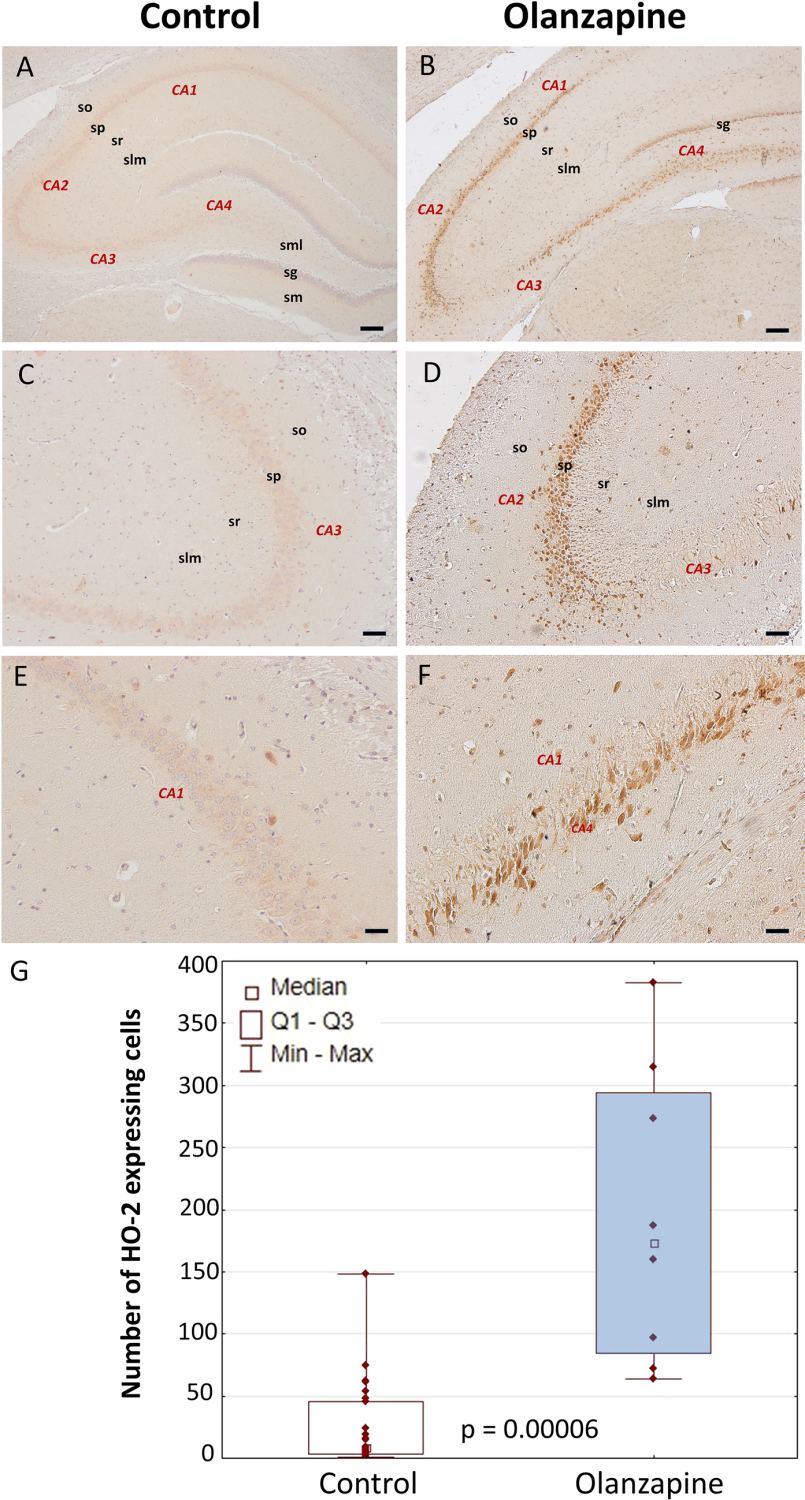
Cellular expression of HO-2 in the hippocampus. An overview (**A**, **B**). Area CA1 (**C**–**F**). DG, dentate gyrus; sg, granular layer of dentate gyrus; slm, stratum lacunosum–moleculare; sm, stratum moleculare of dentate gyrus; sml, multiform layer of the dentate gyrus; so, stratum oriens; sp, stratum piramidale; sr, stratum radiatum. The number of HO-2-expressing cells in the hippocampus of control rats and animals chronically treated with olanzapine (**G**). Data are presented as median with interquartile ranges. Differences between groups (*n* = 5) were statistically analyzed using the Mann–Whitney *U* test and were considered significant at *p* <0.05. Scale bars: 200 μm (**A**, **B**), 100 μm (**C**, **D**), 50 μm (**E**, **F**)

In the hippocampal formation of control rats, there were numerous, multipolar nerve cells with trace or very weakly manifested diffuse expression of HO-2, and single cells with a moderate or moderately intense reaction (Fig. 3 and Supplementary Data). They occurred along the entire length of the pyramidal layer of the horn of Ammon, both in CA1 and CA3, where they were located in a dispersed manner between the pyramidal cell bodies. Very strongly HO-2-positive neurons with highly differentiated cytoarchitecture were scattered throughout the whole striatum. Olanzapine: In the structure of the hippocampal formation, there were very numerous, densely located multipolar perikarya with a remarkably intense, granular expression of HO-2. They occur along the entire length of the pyramidal layer of the horn of Ammon, both in CA1 and CA3, where they are located in a dispersed manner between the pyramidal cell bodies (Fig. 3 and Supplementary Data). They are also numerous cells in the CA4 area and the granular and polymorphous layers of the dentate gyrus. In the structure of the initial layer stratum oriens and the stratum lacunosum–moleculare, there are a few, loosely scattered, small, multipolar HO-2-positive perikarya. Scattered, highly HO-2-positive neurons with highly differentiated morphology were also present in the striatum. Quantitative morphological analysis revealed a very significant increase in the number of hippocampal cells with intense HO-2 immunoreaction in comparison with the control group (*p* = 0.00006, *U* = 5, *N* = 35) (Fig. 3 and Supplementary Data). There were not any statistically significant differences in the striatum (Supplementary Data).

## Discussion

The presented study reveals a statistically significant decrease in the number of CBS-expressing astrocyte-like cells in the striatum and hippocampus of animals chronically exposed to olanzapine. CBS-expressing neurons and glial cells were localized in most regions of the rat brain but the highest number of enzyme-positive neurons were found in the hippocampus and cerebellum [14]. The discussion of the obtained results is particularly difficult, as there are no reports in the literature regarding changes in the expression of enzymes related to the endogenous synthesis of H_2_S in the brain of animals treated with neuropsychiatric drugs. The only point of reference, in this case, is the work of Sommer et al. [15] in which the level of expression of cystathionine β-synthase (CBS), cystathionine γ-lyase (CSE), and mercaptopyruvate sulfotransferase (MPST) was measured in cells of two cell lines: monocytic U-937 and tumor SH-SY5Y (human embryonic neuroblastoma, neuroblastoma) treated with haloperidol, clozapine, olanzapine, and risperidone. For all the antipsychotics used, there was a general decrease in the expression of both CBS and CBE in the cells of the neuronal line. Haloperidol reduced CBS and CSE expression only in SH-SY5Y cells. In turn, olanzapine and risperidone decreased CSE levels in U-937 cells. MPST expression changed only in U-937 cells under the influence of olanzapine, while the administration of clozapine did not result in any effects in both tested cell lines [15]. In general, these results generally correspond to the data obtained in the presented research study, but they do not constitute the basis for formulating binding and unambiguous conclusions, due to the completely different methodology of the conducted research. We are dealing here with the measurement of enzyme expression in cell lines, not in tissue fragments. Second, the changes described in the publication concern neuronal and monocytic cells, not glial cells. The method of administration of drugs was also different. Nevertheless, this publication is valuable and allows for some preliminary considerations regarding the impact of antipsychotics and other pharmaceuticals on the process of endogenous H_2_S synthesis in cells. Several drugs and other substances, such as vitamin D3, can modify the local concentration of H_2_S in a way that depends on the type of cell [16, 17], although the mechanism of these changes is not yet known.

The regulation of gene expression varies in individual tissues, and antipsychotics such as haloperidol and quetiapine may reduce the level of expression of genes encoding antioxidant enzymes [18] and be involved in the formation of H_2_S. It is possible that the inhibitory effect of olanzapine and risperidone on CSE, if present in vivo in humans, may be related to the pharmacological properties of these drugs, both beneficial and potentially undesirable. Of course, hydrogen sulfide is a neuromodulator that is radically different from classic neurotransmitters, primarily because it does not act through the appropriate receptors and classic intracellular signal transduction cascades. Nevertheless, H_2_S may affect the production of local regulatory factors, including cytokines IL-1 and TNF-α [19] and cause various structural modifications of receptor molecules [20], which may be at least theoretically related to the pathogenesis of schizophrenia [19]. It has also been observed that H_2_S has a neuroprotective effect, and induces LTP by increasing the activity of NMDA receptors. The complete elimination of CBS leads to a significant impairment of the LTP generation process [21]. The drug-induced reduction in the number of CBS-positive cells in both the hippocampus and the striatum shown in the study may cautiously suggest a decreased activity of NMDA receptors, which often results in significant deficits in neuroplasticity. Nevertheless, this conclusion is only speculative, as no determinations have been made that could authenticate this hypothesis. Many publications on olanzapine also report its neuroplasticity-promoting properties [22, 23]. However, these studies have mostly been conducted on an induced model of schizophrenia. In turn, post-mortem studies of the brain of schizophrenic patients reveal reduced glutamate levels in the hippocampus and prefrontal cortex, increased expression of the NMDA receptor antagonist kynurenate, and reduced expression of the NMDA receptor NR1 subunit [24]. How this may correspond to changes in signaling associated with local H_2_S synthesis is currently unknown. It was noted, however, that intraventricular administration of homocysteine, a risk factor for the development of Alzheimer's disease, reduces the level of CBS expression in the rat hippocampus with simultaneous impairment of memory and learning. Thus, a reduction in the synthesis of endogenous H_2_S at the level of the horn of Ammon shows a relationship with induced cognitive dysfunctions in rodents [25]. In the normal rat brain, HO-2-expressing neurons were found in the hippocampus, basal ganglia, thalamus, cerebellum, and brainstem [26]. However, the issue of neuronal CO synthesis in the pharmacological aspect is also very poorly researched, which prevents a broader discussion of the significant increase in the number of cells expressing HO-2 in the hippocampus observed in this experiment as a result of chronic exposure to olanzapine. The only publication with which the obtained results can be compared is the work of Chen et al. [27] in which the effect of venlafaxine and the neuroleptic quetiapine on the level of HO-2 expression in the hippocampus of chronically stressed rats was examined. It was observed that the stress-induced reduction of HO-2 expression in hippocampal neurons was significantly minimized by the simultaneous administration of both drugs, while none of them administered individually caused this effect [27]. This may suggest that not only the administration of olanzapine but also the stress-inducing stimuli accompanying the experiment could be one of the reasons for the increased expression of HO-2 in the hippocampus. In patients suffering from neurodegenerative or mental diseases, such as schizophrenia, the expression of HO, and in particular HO-1, is higher in comparison with observed in healthy people and may lead to the impairment of mitochondrial function through improper heme metabolism, and also indicate the presence of oxidative stress. The present study investigated HO-2, a constitutive isoform of HO that, unlike HO-1, is not induced by oxidative stress. HO-2 has proven neuroprotective properties, including by producing CO [28]. One of the physiological effects of CO is the ability to provide adequate circulation necessary for heme detoxification and the stimulation of sGC, which leads to neuroprotective effects [6]. HO-2 also prevents the cytotoxic effects of glutamate and has cytoprotective properties that inhibit apoptosis and reduce oxidative stress caused by TNF-α [28]. The increased number of HO-2 positive cells after administration of olanzapine may, therefore, confirm its potential neuroprotective and pro-neuroplastic effects. We have to point out several limitations of the study. First, there was a small group of rats examined, the gene expressions were not measured and behavioural tests were not yet performed. The conducted experiment is only a modest introduction to further research on the effect of neuropsychiatric drugs on the synthesis of gaseous neuromodulators in animal models with the use of molecular, neurochemical, and imaging tools.

## Conclusions

Olanzapine is a potential regulatory factor of gasotransmitter signaling in the rat brain. Olanzapine may decrease H_2_S synthesis in the astrocytes of the hippocampus and striatum but increase the CO production by Ammon’s horn neurons. Modulatory effect on cellular mechanisms of brain H_2_S and CO synthesis may possibly be an alternative so far unknown way of antipsychotics pharmacological action.

### Supplementary Information

Below is the link to the electronic supplementary material.

Supplementary file1 (PDF 840 KB)

## Data Availability

The data used in the present study are available from the corresponding author upon reasonable request.
