# Supplementary material for: Olanzapine alters the expression of gasotransmitter-related enzymes: CBS and HO-2 in the rat hippocampus and striatum
Source: Pharmacol Rep. 2023 Oct 24;75(6):1610–8. doi: 10.1007/s43440-023-00538-5 (PMC10661766; doi:10.1007/s43440-023-00538-5)
Supplement: Supplementary file 1 — Supplementary file1 (PDF 840 KB) [file 43440_2023_538_MOESM1_ESM.pdf]

Supplementary material

Hippocampus

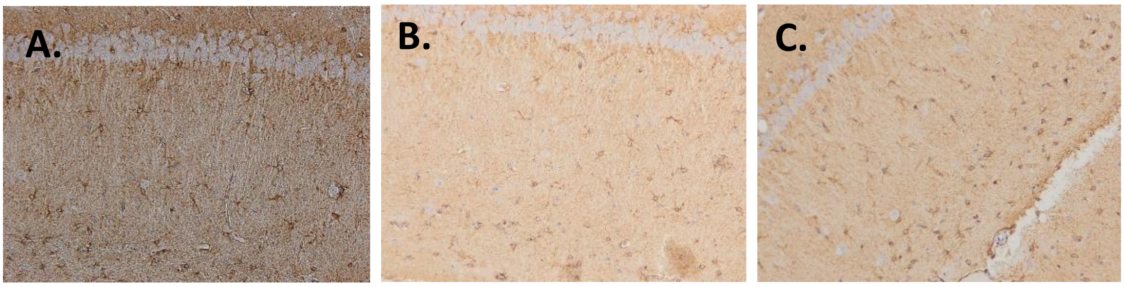

Figure 1. Expression of CBS in the hippocampus. Example images from 3 different animals from control group

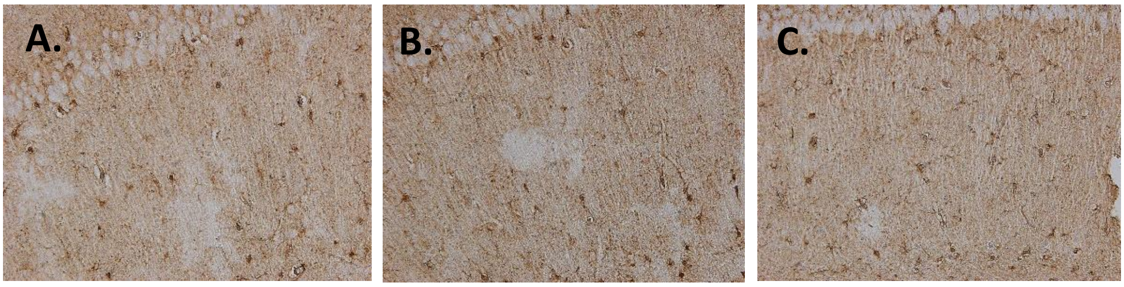

Figure 2. Expression of CBS in the hippocampus. Example images from 3 different animals from experimental group (olanzapine administration).

Striatum

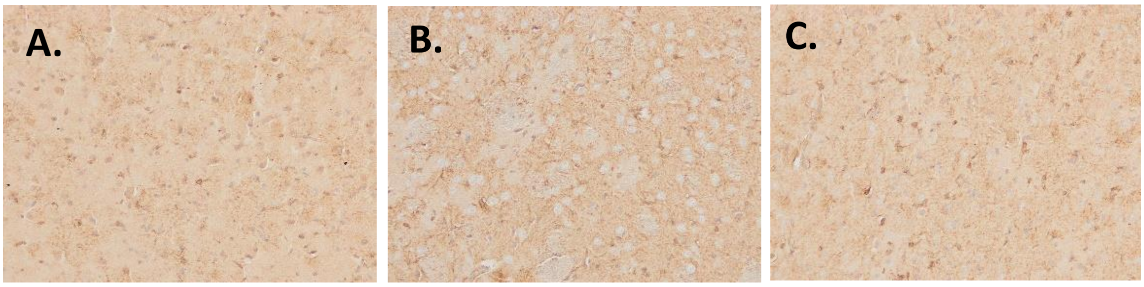

Figure 3. Expression of CBS in the striatum. Example images from 3 different animals from control group

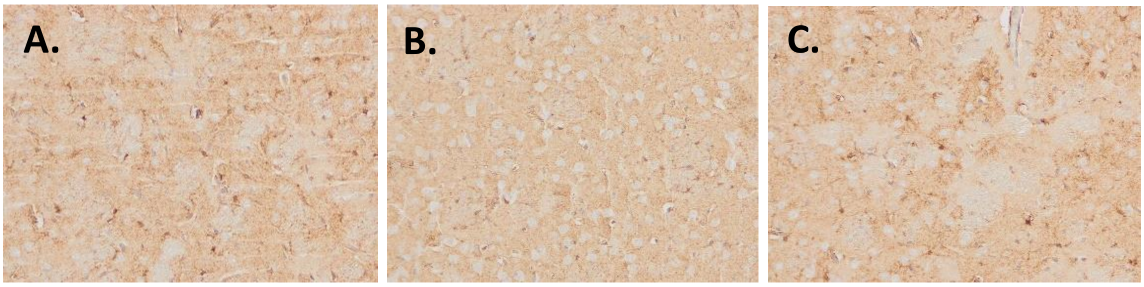

Figure 4. Expression of CBS in the striatum. Example images from 3 different animals from experimental group (olanzapine administration).

Hippocampus

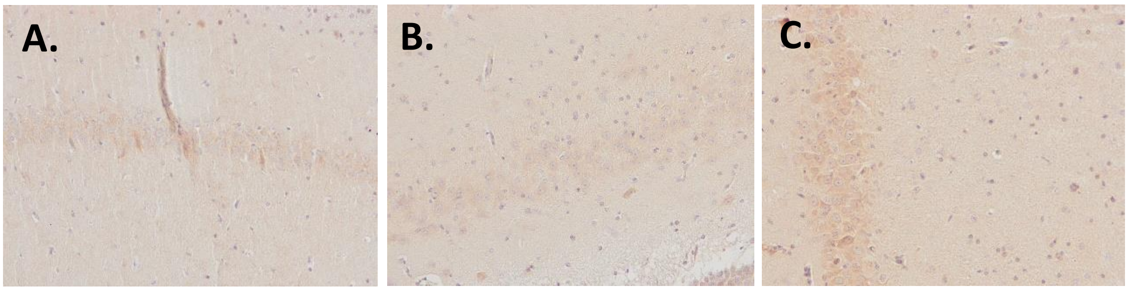

Figure 5. Expression of HO-2 in the hippocampus. Example images from 3 different animals from control group.

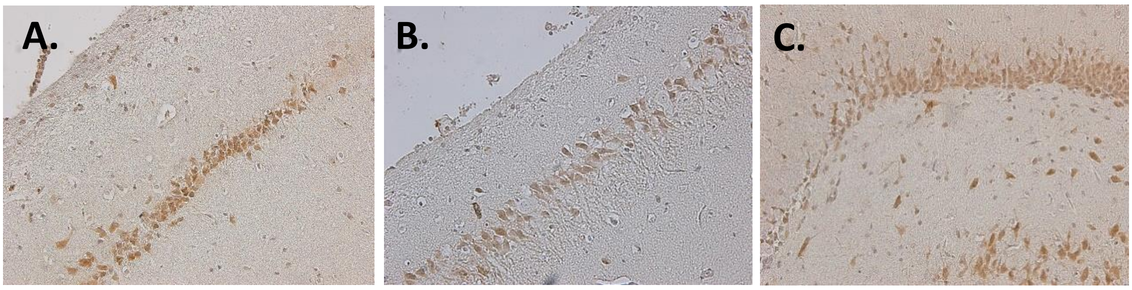

Figure 6. Expression of HO-2 in the hippocampus. Example images from 3 different animals from experimental group (olanzapine administration).

Striatum

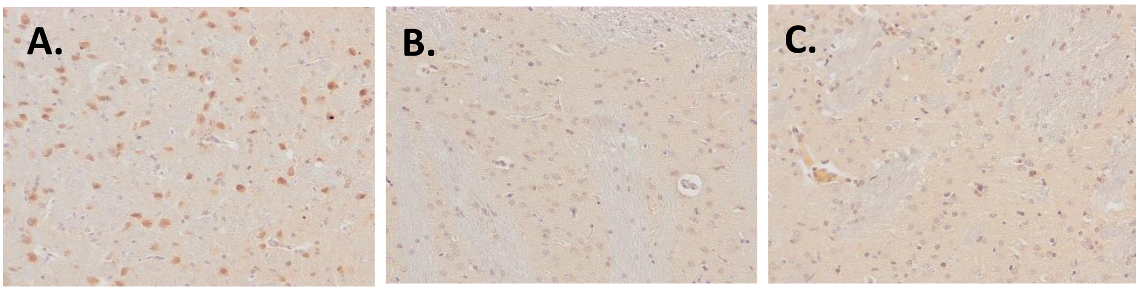

Figure 7. Expression of HO-2 in the striatum. Example images from 3 different animals from control group.

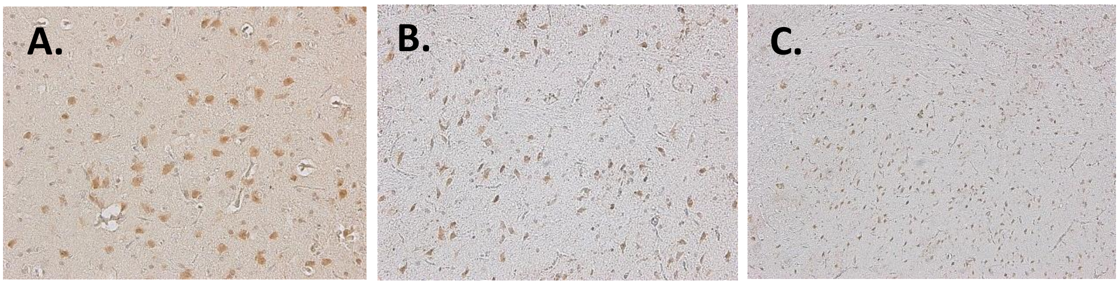

Figure 8. Expression of HO-2 in the striatum. Example images from 3 different animals from experimental group (olanzapine administration).
